# Supplementary figures and images for: Enhancing the therapeutic potential of P29 protein-targeted monoclonal antibodies in the management of alveolar echinococcosis through CDC-mediated mechanisms
Source: PLoS Pathog. 2024 Aug 23;20(8):e1012479. doi: 10.1371/journal.ppat.1012479 (PMC11376570; doi:10.1371/journal.ppat.1012479)

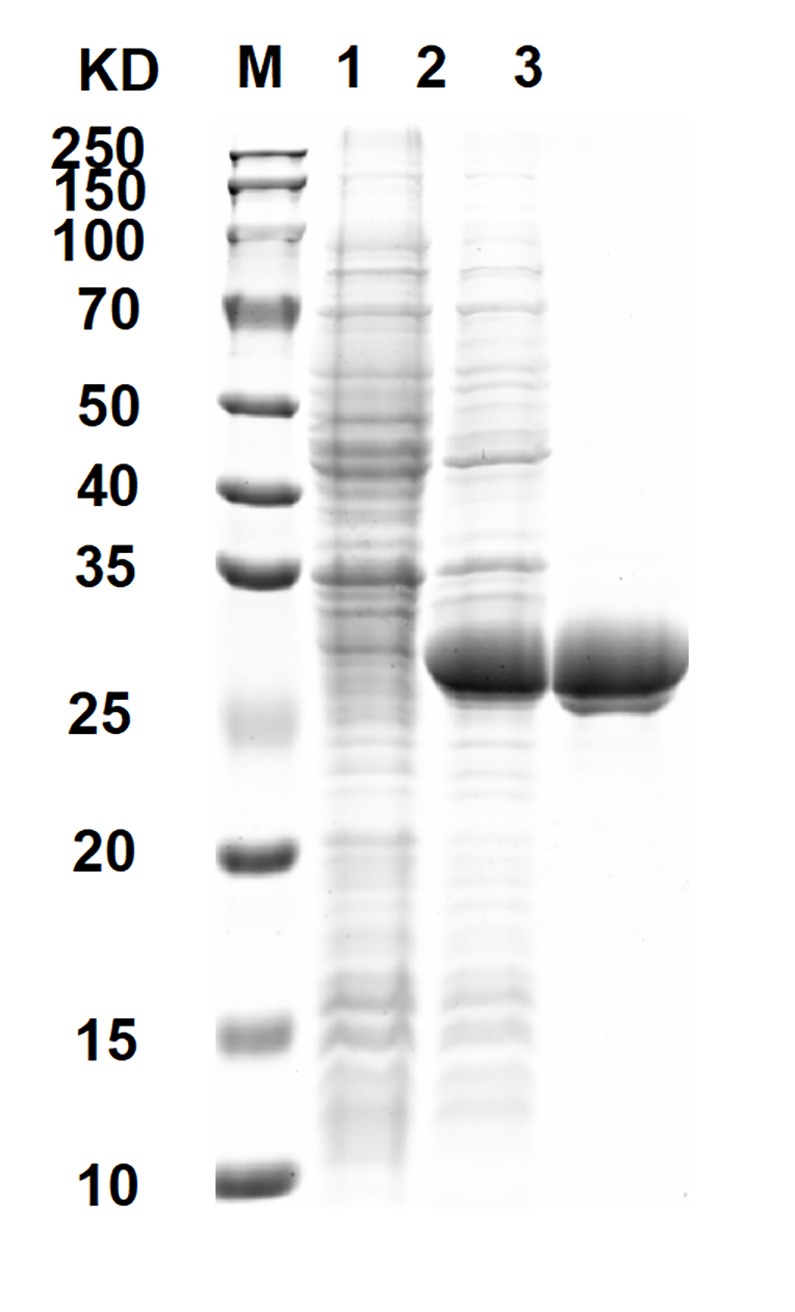

Supplement: S1 Fig — Lane M: protein marker; Lane 1: Escherichia coli containing pET28a-P29 before IPTG induction; Lane 2: E. coli containing pET28a-P29 6h after IPTG induction; Lane 3: purified rEg.P29 using His-affinity chromatography. (TIF) [file ppat.1012479.s002.tif]

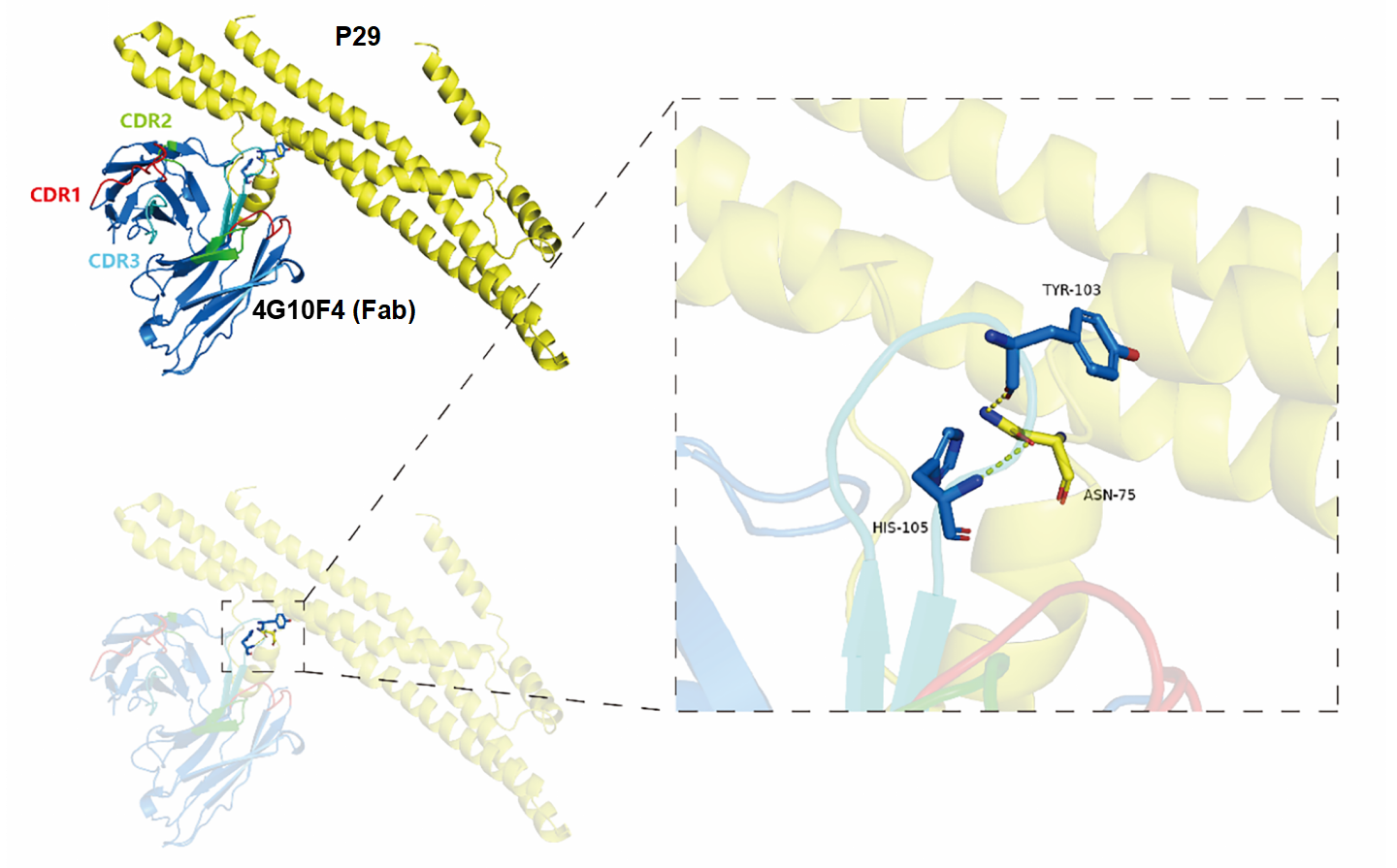

Supplement: S2 Fig — The antibody (blue protein) and P29 (yellow protein) form hydrogen bonds through amino acid residues such as TYR-103, HIS-105 and ASN-75 (yellow dashed line). (TIF) [file ppat.1012479.s003.tif]

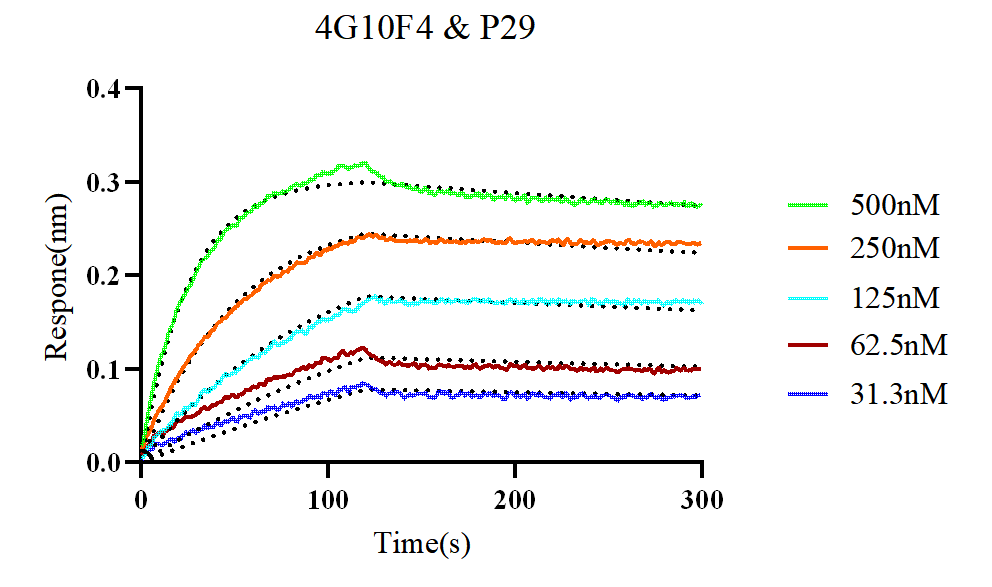

Supplement: S3 Fig — Antigen P29 was diluted to six concentrations (500 nM, 250 nM, 125 nM, 62.5 nM, 31.3 nM, and 0 nM). (TIF) [file ppat.1012479.s004.tif]

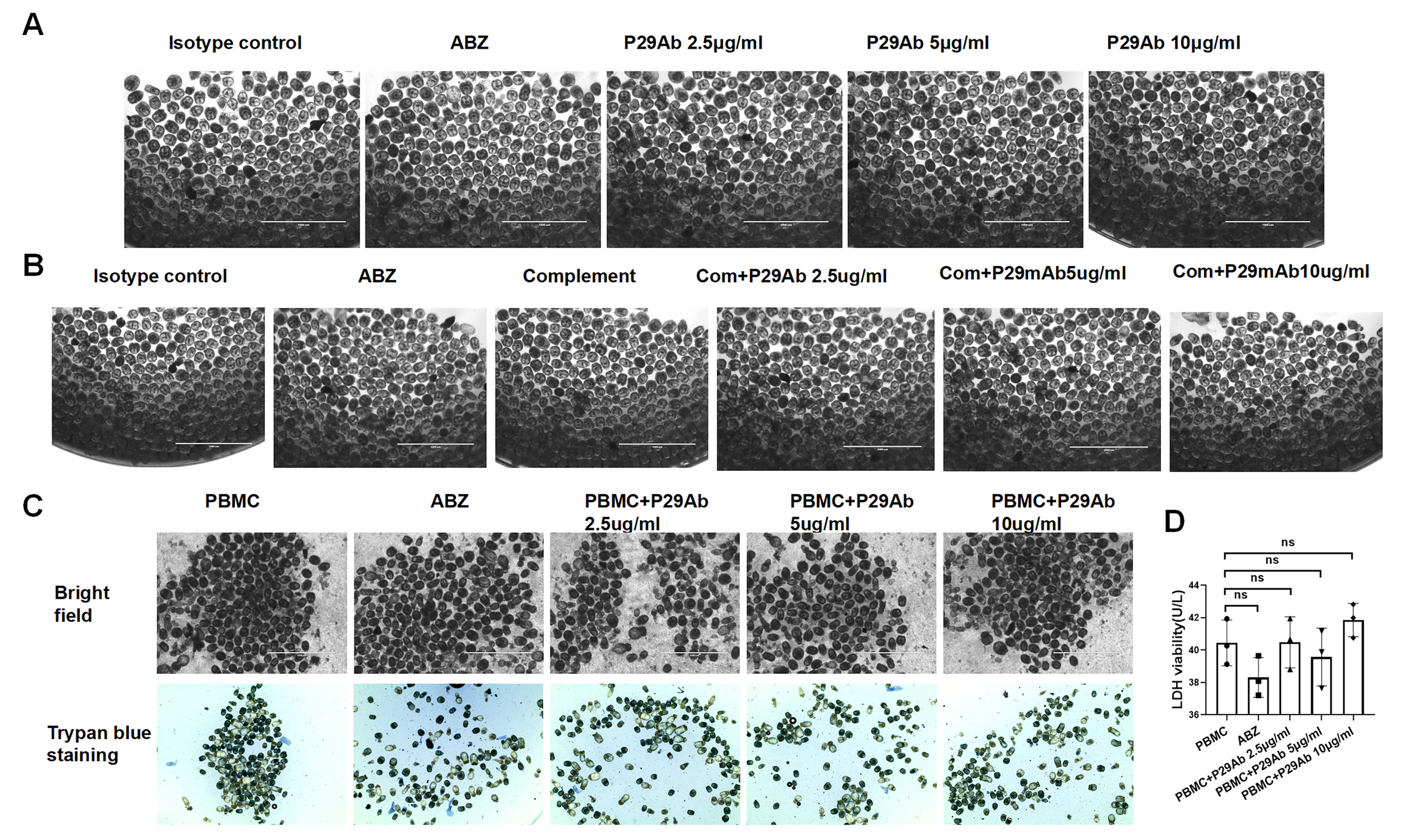

Supplement: S4 Fig — (A, B, C) The morphological characteristics of protoscoleces were examined using an optical microscope 48 hours post-intervention. Upon visual inspection, no discernible variations in protoscoleces were observed between the different groups. (D) Comparion of LDH activity in protoscoleces culture supernatant of different treatment groups. ns: no statistical significance. (TIF) [file ppat.1012479.s005.tif]

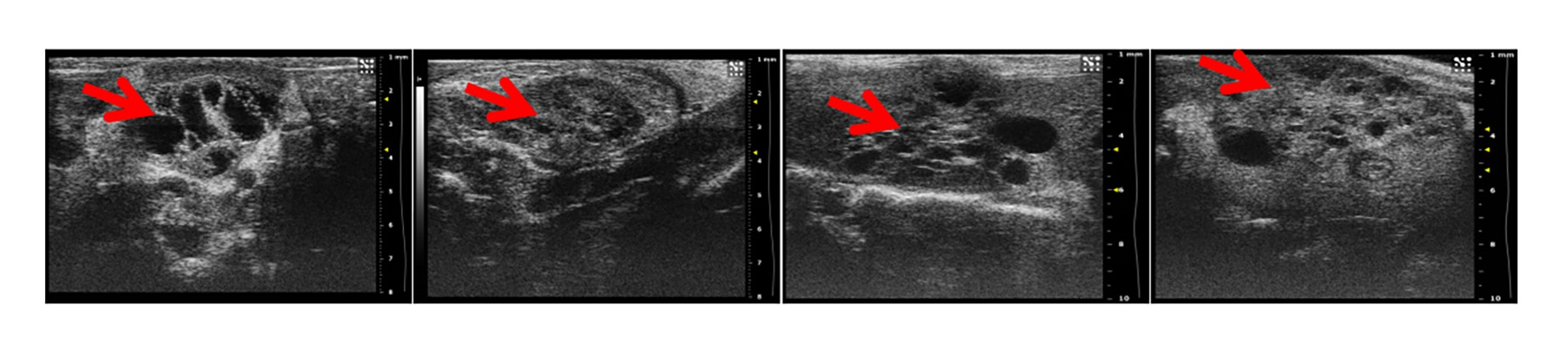

Supplement: S5 Fig — The abdominal ultrasound images revealed the presence of multilocular cystic masses, identified as metacestode lesions, characterized by the presence of echogenic thin septa, as denoted by the red arrows. (TIF) [file ppat.1012479.s006.tif]

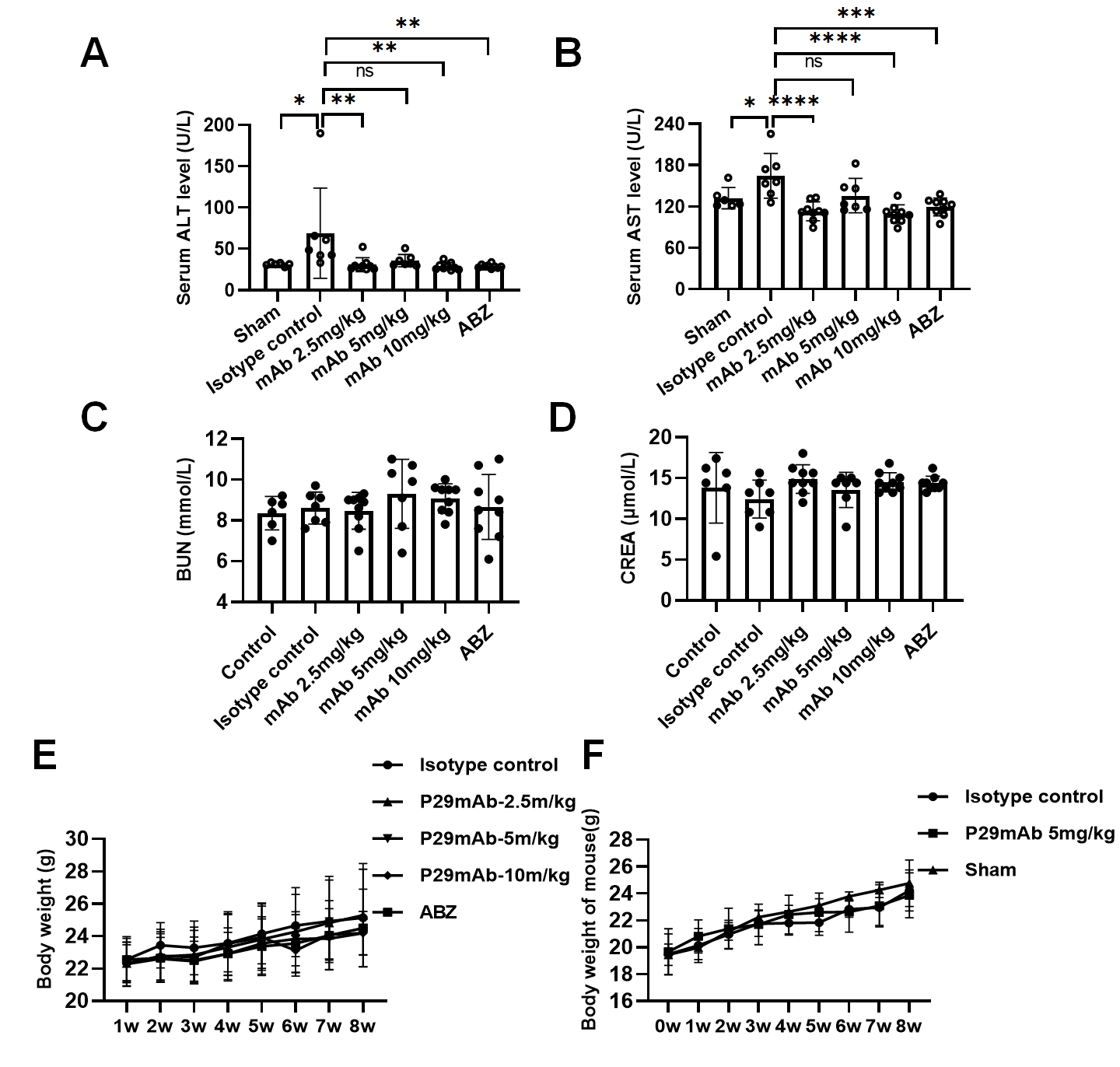

Supplement: S6 Fig — (A-F) IL-6, IL-10, MCP-1, IFN-γ, TNF, and IL-12p70, were compared between groups in an intraperitoneal infected mice model following treatment. (TIF) [file ppat.1012479.s007.tif]

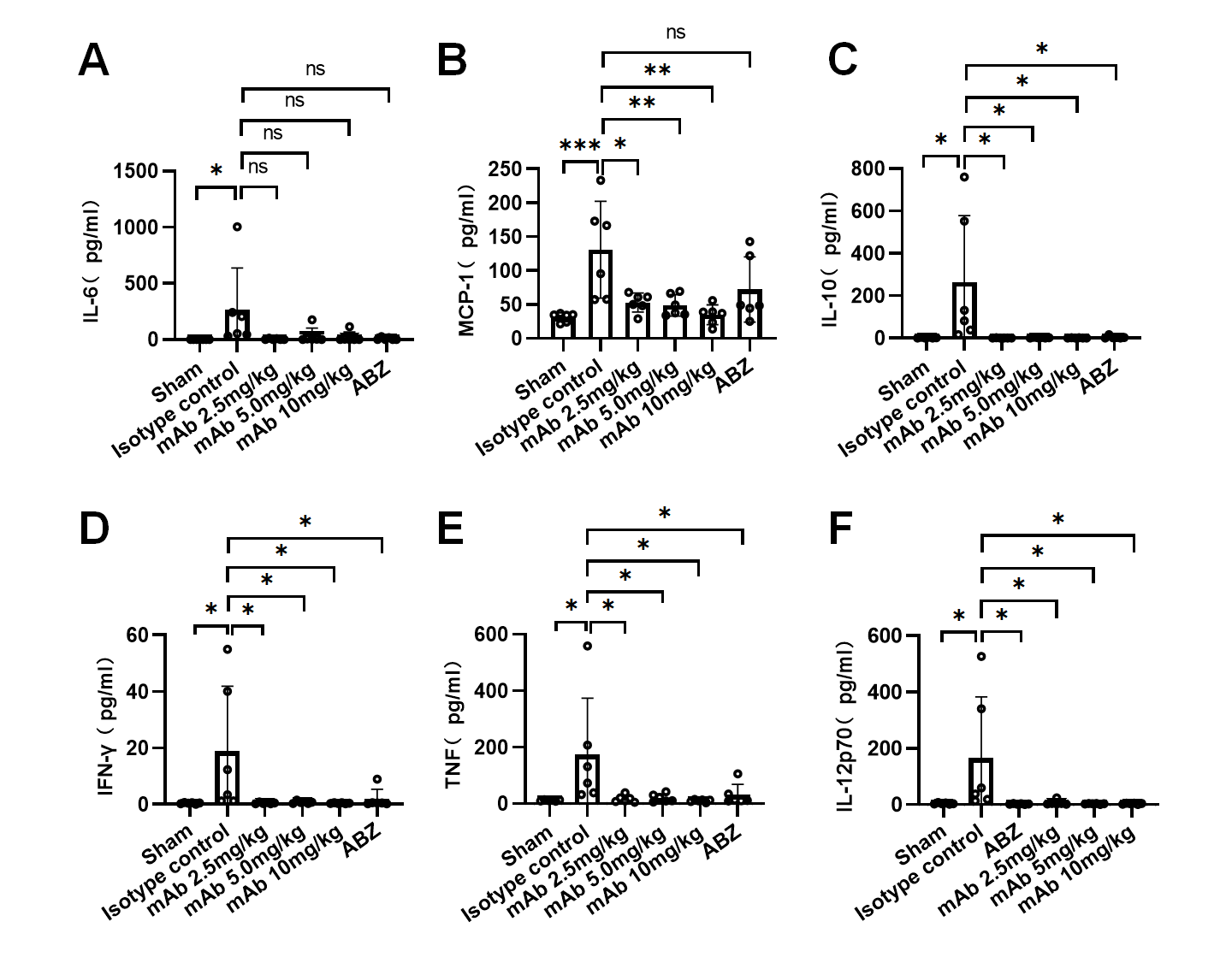

Supplement: S7 Fig — (A,B) Comparison of alanine aminotransferase (ALT) and aspartate aminotransferase (AST) serum concentrations between groups after treatment. (C and D) The concentrations of blood urea nitrogen (BUN) and creatinine (CREA) were analyzed between groups following treatment in an intraperitoneal infected mice model. (E) The body weight was monitored throughout the course of the experiment involving the treatment of intraperitoneal infected mice. (F) The body weight was monitored during the experiment involving the treatment of mice infected through the portal vein. (TIF) [file ppat.1012479.s008.tif]
