# Supplementary material for: Enhancing the therapeutic potential of P29 protein-targeted monoclonal antibodies in the management of alveolar echinococcosis through CDC-mediated mechanisms
Source: PLoS Pathog. 2024 Aug 23;20(8):e1012479. doi: 10.1371/journal.ppat.1012479 (PMC11376570; doi:10.1371/journal.ppat.1012479)
Supplement: Attachment — Submitted filename: Authors Responses to Editor and Reviewers Comments.docx [file ppat.1012479.s015.docx]

Listed Responses to the Editor and Reviewers

Dear Editors and Dear Reviewers:

Thank you for your efforts to review our paper (PPATHOGENS-D-24-00162) entitled: “Enhancing the Therapeutic Potential of P29 Protein-Targeted Monoclonal Antibodies in the Management of alveolar echinococcosis through CDC-Mediated Mechanisms”. We have revised the manuscript based on your valuable suggestions. Followings are listed responses to your comments and suggestions:

**Major Issues: Key Experiments Required for Acceptance**

**Reviewer #1: 1.** **In figure 2D, what does the highest peak next to the Blank group represent?**

Response: Thanks for your critical question. In figure 2D, the X-axis data in the original graphic is obscured due to width restrictions. We have modified the graph to display the complete dataset. The highest peak adjacent to the blank group corresponds to the r*Eg*P29 sample (positive control).

**2. Please provide deeper insights into the mechanism of action of 4G10F4, especially its interaction with the immune system beyond CDC.**

Response: Thanks. This problem is of great concern to us. Our in vitro experiments revealed the CDC action of 4G10F4. In in vivo experiments conducted on mice, we noted that treatment with 4G10F4 significantly reduced the levels of the six inflammatory markers when compared to non-infected control mice. This observation suggests potential anti-inflammatory properties induced by the monoclonal antibody targeting P29. Nonetheless, the precise functions of P29 remain unclear, necessitating further research to elucidate the specific underlying mechanisms.

**3.** **What are the long-term effects and safety of** **4G10F4 treatment? If the authors have done any extend research for evaluation?**

Response: This study presents an initial assessment of the potential anti-*E.multilocularis* activity of 4G10F4 in infected mice, with observations indicating no adverse effects on body weight, liver and kidney function, or mortality during the treatment period. However, further investigation into the long-term effects and safety profile of 4G10F4 treatment is warranted, necessitating additional research in the future.

**Reviewer #2: 1. My main concerns with the experimental data center around Figure 4. Specifically, where in the parasite do these primary cells originate from, and can you confirm these cells actually express P29? P29 expression within the parasite appears to be tissue-specific, as it is absent from cyst fluid, so it is possible that the primary cells do not express it, or do not express it uniformly. An immunoblot of primary cell lysates probed with the 4G10F4 mAb would partly address this concern. It would also be helpful to know what percentage of cells express P29, and where in the cells the P29 antigen is located - e.g. is it membrane-associated? Is it expressed on the cell surface? Or in intracellular vesicles? Perhaps some immunofluorescence microscopy would be appropriate to address these concerns.**

Response: The primary cells were derived from in vitro cultured metacestode vesicles, as outlined in the Methods section "Isolation of E. multilocularis primary cells". The detailed culture process of primary cells is further elucidated in our previously published article[1].

In supplementary experiments, *Em* tissue sections were utilized to evaluate the expression of P29 through immunofluorescent staining with 4G10F4 as the primary antibody. The results depicted in **r.Figure 1** showed red fluorescence indicating the presence of P29 protein and blue fluorescence indicating cell nucleus. P29 protein was observed to be widely distributed in the germinal layer of the parasitic vesicles and tegument of protoscolices. To validate the expression of P29 in primary cells, immunofluorescence staining was conducted using 4G10F4 as the primary antibody targeting P29. The results depicted in **r.Figure 2** indicate that green fluorescence corresponds to the presence of P29 protein, while blue fluorescence indicates the cell nucleus. The localization of P29 was observed in both the cytoplasm and cell membrane. Unfortunately, due to the limited number of metacestode vesicles cultured in vitro at this stage, fewer cells were the isolated, thus hindering the accurate calculation of the percentage of cells expressing P29. Nevertheless, based on the immunofluorescence staining results of *Em* tissue, widespread presence of P29 was noted. Future research endeavors will aim to enhance the precision of this experiment in order to elucidate the proportion of cells expressing P29.

**2. The in vivo experiments are a strength of the manuscript, but it is important to show these results are reproducible. At a minimum, the authors should show that the results have been recapitulated in two independent experiments.**

Response: Thanks. The animal experiment for intraperitoneal infection was conducted three times, while the experiment for portal vein infection was repeated twice due to the complexity of the procedure. The results of these experiments consistently demonstrated that 4G10F4 effectively reduced parasite burdens in mice infected with E. multilocularis protoscoleces via both intraperitoneal and portal vein routes. The results from the repeated experiments were presented in **rFigure 3,** **rFigure 4 and rFigure 5**.

**3.An ethics statement, indicating the animal experimentation was reviewed and approved by an appropriate institutional review committee before the work was undertaken, is required.**

Response: Thanks for your advice. In the “Supplementary figures and data”, we provide the ethical proof of animal experiments- “Affidavit of Approval of Animal Ethical and Welfare” showed in r.Figure 7.

**Reviewer #3: - The impact of mAb treatment on the development of the metacestode stage development should be also assessed when mice are treated 1 week after intraperitoneal infection. Similarly, given that metacestode tissue can be easily cultured and produces vesicles in vitro, it should be tested whether the mAb (+/- complement) prevents the release of vesicles in vitro. Optimally, you could** **infect mice/gerbils with these in vitro treated metacestodes to confirm that the treatment prevents the establishment of new infections / spreading** **of the metacestode stage.**

Response: Thanks for your suggestion. In our prior experiments, utilizing mouse models infected through intraperitoneal injection, we observed a success rate of approximately 60% in establishing infection, suggesting a failure rate of approximately 40%. To ascertain the specific therapeutic impact of 4G10F4 against E. multilocularis in vivo, we opted to commence antibody intervention 12 weeks post-infection, at which point lesions were visible. In future research, the early intervention effect of antibody therapy on the metacestode can be assessed by treating mice one week after intraperitoneal infection, with an expanded sample size to mitigate errors resulting from modeling failure.

In our research, we conducted experiments to assess the impact of mAb with or without complement on metacestode vesicles cultured in vitro. The findings, presented in **rFigure 6**, indicated that mAb (with or without complement) did not affect the structural integrity or growth of the vesicles. This observation suggests that the protective laminated layer , a carbohydrate-rich acellular structure surrounding the parasitic vesicles [2], may impede the interaction between antigen P29 and antibodies by limiting antigen exposure. The therapeutic action of the monoclonal antibody on *Echinococcus multilocularis* vesicles in vivo may be attributed to the presence of abundant immune cells, including macrophages and neutrophils, which have the capacity to disrupt the barrier integrity of the laminated layer surrounding metacestode vesicles through the secretion of specific enzymes. This process facilitates antigen exposure and enhances the efficacy of antibody treatment. As per your suggestion, future research endeavors may involve infecting mice or gerbils with in vitro treated metacestodes to ascertain whether the monoclonal antibody treatment effectively inhibits the dissemination or proliferation of the metacestode.

**- Fig. 2: Given that you are focusing in this manuscript on E. multilocularis, the binding of the mAb to vesicles and metacestode tissues of E. multilocularis should be shown as well.**

Response: Thanks for your suggestion. *Em* tissue sections were utilized to evaluate the expression of P29 through immunofluorescent staining with 4G10F4 as the primary antibody. The results depicted in **r.Figure 1** showed red fluorescence indicating the presence of P29 protein and blue fluorescence indicating cell nucleus. P29 protein was observed to be widely distributed in the germinal layer of the parasitic vesicles and tegument of protoscolices.

**- Does the mAb (+/- PBMCs, complement) impact the protruding of the protoscolices?**

Response: No. The morphology and protruding of the protoscolices were not affected by the in vitro intervention of mAb(+/-PBMC, complement) or ABZ when observed under the microscope.

**Part III – Minor Issues: Editorial and Data Presentation Modifications**

**Please use this section for editorial suggestions as well as relatively minor modifications of existing data that would enhance clarity.**

**Reviewer #1: 1. Figure 2E, please label clearly the species of P29 and clarify how** **the putative three-dimensional structures of P29 and the anti-P29 antibody were obtained. Please also label the** **CDRs on the antibody structure. If they are also close to the epitope on P29? Also, please provide a complete protein sequence for the antibody in addition to the gene sequencing results provided, which will be helpful to better understand the protein feature instead of searching multiple sequencing results.**

Response: Thanks. We have labeled the species of P29 (*Eg* P29) in revised Fig 2E legend, and provided the method for predicting the three-dimensional structures of P29 and the anti-P29 antibody (Line185). CDRs were labelled on the antibody structure as shown in revised Fig 2E, and close to the epitope we screened on P29. The complete protein sequence for 4G10F4 mAb was provided in **r.Table 1**.

**2. SPR binding curve should be remade by using the exported raw data instead of a screenshot.**

Response: Thanks. We have provide the SPR binding curve by using the exported raw data instead of the screenshot in revised S3 Fig.

**Reviewer #2: Line 157 and Fig 1C - what is the relevance of titers here? In what source material?** **Hybridoma supernatant? If so, the "titer" will depend on variables related to culture of the hybridoma cells, which is not really relevant. Unless you normalized the amount of each antibody in this analysis, I am not certain the data add anything to the manuscript.**

Response: Thank you for your insightful inquiry. In this study, the hybridoma culture supernatant was used for antibody titer detection. We quantified the protein concentration of the culture supernatant from the five hybridoma cell lines using the BCA method, and subsequently normalized the protein levels for titer analysis and westernblot analysis. A clarification regarding this procedure has been included in the revised manuscript (Line 157).

**Fig.1D - not sure the results from this experiment can be interpreted as quantitative - I would avoid that language at line 159 etc.**

Response: Thanks. We have revised the sentence in our manuscript. (Line 161)

**Fig. 2B, 2C - is the parasite material E. granulosus or E. multilocularis? Please clarify**

Response: Thanks. In Fig 2B and Fig 2C, we examined the specific binding of mAb 4G10F4 to *echinococcus granulosus* materials. We have clarified it in the revised manuscript. (Line 166)

**Line 186 - "SPR" - please define (presumably** **surface plasmon resonance, but please clarify to avoid ambiguity)**

Response: Done. Thanks. (Line 196)

**Fig 3 - why use three different methods to assess protoscolex viability? Consistency in methodology would have allowed a direct comparison between these data. As it stands, I'm not sure how they compare. Was an isotype control antibody with irrelevant specificity used as the negative control?**

Response: Thanks for your critical question. In Fig 3, we used eosin and trypan blue staining techniques, alongside CCK8 cytotoxicity detection methods to assess the viability of protoscoleces. Eosin and trypan blue staining are commonly utilized approaches for quantifying the viability proportion of protoscoleces, by determining the ratio of deceased to living protoscoleces [8]. Here we used it to determine whether PSCs viability was greater than 95% and can be used in subsequent intervention experiments. The CCK8 assay was used to assess the PSC viability in mAb direct intervention test and CDC test because of its convenient methodology and impartiality. In ADCC test, because of PBMC interference, we used trypan blue staining. In this section, isotype-mouse IgG1 antibody was used as negative control and ABZ was used as positive control. It was clarified in the revised manuscript (Line 709).

**Fig. 5 - sham data for C and D would be helpful to see. Fig. 5G-5L - what is the "blank" group?**

Response: Thanks for your critical question. We have added sham data to Fig 5C and Fig 5D. “Blank” group means “Sham” group. To avoid any potential confusion, we have rectified the labeling from "Blank" to "Sham" in Figures 5G-5L.

**Line 329 - "CE2"?**

Response: Based on the ultrasonographic features, cystic echinococcosis (CE) are differentiated into five types using the WHO IWGE standard: CE1, CE2, CE3 (a, b), CE4, and CE5. The hydatid cyst type is used to assess their stage of development. Type CE1 and CE2 cysts are considered active cysts and usually fertile with viable protoscoleces [3].

**Discussion - first part is a reiteration of points made in the Introduction. Discussion could be made more concise elsewhere too, e.g. there is no need to provide a detailed discussion of antibody structure and function, as this is well established.**

Response: Thanks. We have concised some parts of the discussion including the first part.

**Reviewer #3: - The different impact on the acute versus established infection should be clearly highlighted in the abstract and the discussion.**

Response: Thanks. We have made some amendments in the abstract and discussion sections to highlight the different impact on the two infections.

**- Line 423f: Please show the data that demonstrates that mAb and complement treatment of primary E. multilocularis cells prevented the formation of clones.**

Response: As depicted in Fig 4A, the control and antibody treated groups exhibited discernible cloned cell clusters formed by E. multilocularis primary cells, whereas the ABZ and complement treated groups displayed a reduction in the size of these cell clusters. Notably, the anti-P29 mAb plus complement treated group demonstrated a near absence of clones formed by the *E. multilocularis* primary cells. We added red circles to highlight the cell clones in Fig4A. We can only obtain qualitative analysis data in Fig 4A, unfortunately, we can't do a good quantitative analysis.

**- Please discuss your reasoning of the selected treatment regimen (once per week).**

Response: Monoclonal antibodies demonstrate a slow tissue penetration rate and extended serum half-lives, typically lasting from days to weeks[4]. Consequently, the standard protocol for monoclonal antibody therapy involves administration once or twice weekly[5-7]. In this study, we opted to administer the mAb once per week as a cost-saving measure. In future studies, we will explore the efficacy of administering the mAb twice weekly to determine an optimal treatment protocol.

**- Please discuss and reference the potential use of IgG1 versus IgG2 monoclonal antibodies for therapeutic approaches.**

Response: Thanks. We have added the discuss and reference the potential use of IgG1 versus IgG2 monoclonal antibodies for therapeutic approaches in revised manuscript.(Line 358-368)

**- The description of the in vitro and in vivo results should be rephrased in the abstract. Clearly indicate that CDC is required for mAb efficacy in vitro and highlight the differences in both in vivo experiments.**

Response: Thanks. We have made some amendments in the abstract as your suggestion.

**- As the methods section is at the end of the article, it is necessary to shortly introduce some methods/readouts already in the results section (as mentioned below).**

**- Please check the English by a native speaker.**

Response: Thanks. A native speaker has been invited to help improve the language.

**- Line 96: “Clostridium difficile” in italics**

Response: Done. Thanks.(Line 96)

**- Fig. 1 B, C: Please use colors to facilitate the differentiation of the different IgG classes / clones.**

Response: Done. Thanks.

**- Fig. 2B: Line 164 to 165. Please delete the sentence “The purified mAb exhibited …” and only state that the mAb binds to PSC and HCW, but not HCF, as you did in the following sentence.**

Response: Done. Thanks.(Line 166-168)

**- Fig. 4: Please explain** **the origin of E. multilocularis primary cells already in the results section. Please increase the size of Fig. 4A, as it is not possible to detect the described differences.**

Response: Thanks for your suggestion. We have added the explanation for the origin of *E. multilocularis* primary cells in the results section (Line 229). And we increased the size of Fig 4A as well as circled the cell clones to highlight the differences between groups.

**- Line 235: please indicate the treatment regimen used.**

Response: Done. Thanks.

**- Figure 6 and 8 legend: indicate the number of mice used per group and number of repeat experiments and whether the mean or median is shown.**

Response: Thanks. The number of mice used per group was indicated in Fig 6 and Fig 8. The animal experiment for intraperitoneal infection was repeated three times, while the experiment for portal vein infection was repeated twice due to the complexity of the procedure. The results from the repeated experiments were consistent with Fig 6 and Fig 8, and presented in **rFigure 3,** **rFigure 4** and **rFigure 5.** The data shown was a comparison of the mean of each group.

**- Line 243: Please delete the sentence “A similar effect was seen…”. This is not supported by the shown data.**

Response: Thanks. The sentence was revised.(Line 264)

**- Line 247f.: Please explain shortly in the results section how you calculate the spleen and liver index.**

Response: Thanks. Done.(Line 269)

**- Line 249ff: How did you assess the metacestode inhibition rates and where is this data shown? Do you refer to Fig. 6B? Please clarify.**

Response: Thanks. It was described in methods section as “The metacestode inhibition rate (%) was calculated as: (total wet weight of metacestode tissue in control group - total wet weight of metacestode tissue in treatment group) / total wet weight of metacestode tissue in control group×100”. The data of wet weights of the metacestode in each group was shown in Fig 6B. Based on the wet weights of the metacestode, metacestode inhibition rates were calculated. And we have clarified it in revised manuscript. (Line 272)

**- I recommend to combine Fig. 5 and Fig. 6A-D into one figure and Fig. 6E-L as additional figure. This way the figures fit to the paragraphs in the results section.**

Response: Thanks. Done as you suggested.

**- Fig. 6G-L: Similar to the ALT and AST values, cytokine levels should be compared to sham mice, not blank controls. The x-axis should not show negative cytokine levels.**

Response: Thanks for your critical question. We have corrected the error in Fig 6G-L.

**- Fig. 8A, B, F: Increase the size of these figures, to facilitate the identification of the metacestodes. E.g., it is currently not possible to identify the PAS-positive sections.**

Response: Thanks for your suggestion. We have increased the size of these figures as much as possible.

**- I recommend to combine Fig. 7 and 8.**

Response: Thank you for your suggestion, with which we concur. However, due to spatial constraints, the integration of figures 7 and 8 into a single layout was not feasible, leading us to maintain them as separate figures.

**- Please rephrase the term “secondary infection”. Clearly state the experimental design (e.g. i.p. infection with protoscolices followed by i.v. mAb treatment)**

Response: The experimental induction of echinococcosis can be categorized into primary and secondary infections. Primary infection involves the oral administration of hydatid eggs to mice, whereas secondary infection entails the inoculation of mice with hydatid metacestode.

The treatment regimen was outlined in the methods section, wherein mice were intraperitoneally infected with 2000 protoscolices and subsequently treated with mAb at doses of 2.5, 5, and 10 mg/kg via tail vein injection once weekly for 8 weeks at 12 weeks post-infection. Mice infected with 1000 protoscolices via portal vein injection were treated with mAb at a dose of 5 mg/kg via tail vein injection once weekly for 8 weeks at one week post-infection.

**- Line 327: add reference**

Response: Thanks. Done. (Line 344)

**- Line 397-410: I recommend to mention this shortly in the results section (instead of the discussion) to explain the selection of the used methods.**

Response: Thanks. We have revised the manuscript as your suggestion.

Figures have been processed by PACE digital diagnostic tool to meet PLOS requirements. Excel spreadsheet with numerical data for Figure panels 1B, 1C, 2D, 3B, 3C, 3D, 4B, 4D, 5C, 5D, 5E, 7C, 7D and 7E was provided in Data S1.

r.Figure 1: Confocal microscopy image of the *E. multilocularis* tissue P29 recognized by 4G10F4 mAb.

r.Figure 2: Confocal microscopy images of the *E. multilocularis* cells P29 recognized by 4G10F4 mAb.

r.Figure 3: Repeated experiment: evaluation of therapy effects of 4G10F4 on *E. multilocularis* intraperitoneal infected mice (n=8/group).

r.Figure 4: Repeated experiment: evaluation of therapy effects of 4G10F4 on *E. multilocularis* intraperitoneal infected mice (n=10/group).

r.Figure 5: Repeated experiment: evaluation of therapy effects of 4G10F4 on *E. multilocularis* infected mice through portal vein (n=5/group).

r.Figure 6: Effects of 4G10F4 on *E. multilocularis* derived daughter vesicles in vitro.

r.Figure 7: Affidavit of Approval of Animal Ethical and Welfare.

r.Table 1: Complete protein sequence for 4G10F4 mAb.

Thanks again to the editor and reviewers for their efforts to help improve the quality of this paper.

**r.Figure legends**

**r.Figure 1**: Confocal microscopy image of the ***E. multilocularis* tissue** P29 recognized by 4G10F4 mAb. Red fluorescence indicating the presence of P29 protein and blue fluorescence indicating cell nucleus. PV: parasitic vesicle; GL: germinal layer; PSC: protoscolex.

**r.Figure 2**: Confocal microscopy images of the ***E. multilocularis* cells** P29 recognized by 4G10F4 mAb. Green fluorescence indicating the presence of P29 protein and blue fluorescence indicating cell nucleus.

**r.Figure 3**: Repeated experiment: evaluation of therapy effects of 4G10F4 on *E. multilocularis* intraperitoneal infected mice (n=8/group). (A) Gross morphology of metacestode lesions. (B) Quantitative weight analysis of the E.multilocularis metacestode tissues. *p < 0.05, ns: no statistical significance.

**r.Figure 4**: Repeated experiment: evaluation of therapy effects of 4G10F4 on *E. multilocularis* intraperitoneal infected mice (n=10/group). (A) Gross morphology of metacestode lesions. (B) Quantitative weight analysis of the E.multilocularis metacestode tissues.

**p < 0.01, ns: no statistical significance.

**r.Figure 5**: Repeated experiment: evaluation of therapy effects of 4G10F4 on *E. multilocularis* infected mice through portal vein (n=5). (A) Gross morphology of metacestode lesions in liver. (B) Comparison of the numbers of infectious foci in the liver surface between groups after treatment. ***p < 0.001.

**r.Figure 6**: Effects of 4G10F4 on *E. multilocularis* derived daughter vesicles in vitro. (A) Light microscopical images of vesicles cultivated on day 0 and day 8 in different groups. The vesicles in ABZ-treated group exhibited collapse, while vesicles in other groups remained intact and increased in size. (B) Comparison of volumes of vesicles cultivated on day 0 and day 8 of each group. ns, not significant.

**r.Figure 7**: Affidavit of Approval of Animal Ethical and Welfare.

**r.Table 1. Complete protein sequence for 4G10F4 mAb.**

| **Chain Type** | **Sequence** |
| --- | --- |
| **H** | EVKLEESGPGLVAPSQSLSITCTVSGFSLFSYAVHWVRQPPGKGLEWLGVIWAGGSTDYNSALMSRLSISKDNSKSQVFLKMNSLQTDDTAMYYCASIHYYGYWHFDVWGAGTTVTVSSAKTTPPSVYPLAPGSAAQTNSMVTLGCLVKGYFPEPVTVTWNSGSLSSGVHTFPAVLQSDLYTLSSSVTVPSSTWPSETVTCNVAHPASSTKVDKKIVPRDCGCKPCICTVPEVSSVFIFPPKPKDVLTITLTPKVTCVVVDISKDDPEVQFSWFVDDVEVHTAQTQPREEQFNSTFRSVSELPIMHQDWLNGKEFKCRVNSAAFPAPIEKTISKTKGRPKAPQVYTIPPPKEQMAKDKVSLTCMITDFFPEDITVEWQWNGQPAENYKNTQPIMDTDGSYFVYSKLNVQKSNWEAGNTFTCSVLHEGLHNHHTEKSLSHSPGK |
| **L** | DIVMTQSPLSLPVSLGDQASISCRSSQSIVHSNGNTYLEWYLQKPGQSPKLLIYKVSNRFSGVPDRFSGSGSGTDFTLKISRVEAEDLGVYYCFQGSHVPYTFGGGTKLEIKRADAAPTVSIFPPSSEQLTSGGASVVCFLNNFYPKDINVKWKIDGSERQNGVLNSWTDQDSKDSTYSMSSTLTLTKDEYERHNSYTCEATHKTSTSPIVKSFNRNEC |

**References**

[1] Zhang C, Li Z, Fu Y, Li T, Hou S, Wang C, Li M, Zhao W. A SIMPLE, QUICK, AND ECONOMICAL METHOD FOR IN VITRO CULTIVATION OF ECHINOCOCCUS MULTILOCULARIS METACESTODE AND GENERATION OF PRIMARY CELLS. J Parasitol. 2024 May 1;110(3):210-217.
[2] Díaz A, Casaravilla C, Irigoín F, Lin G, Previato JO, Ferreira F. Understanding the laminated layer of larval Echinococcus I: structure. Trends Parasitol. 2011 May;27(5):204-13.
[3] WHO Informal Working Group. International classification of ultrasound images in cystic echinococcosis for application in clinical and field epidemiological settings. Acta Trop. 2003 Feb;85(2):253-61.
[4] Zinn S, Vazquez-Lombardi R, Zimmermann C, Sapra P, Jermutus L, Christ D. Advances in antibody-based therapy in oncology.
[5] Li F, Li J, Yin K, Zhang J, Li ZH, Lu L, Bao YW, Qin Z, Zheng Y, Yang BT, Li J, Wang X. CS1003, a novel human and mouse cross-reactive PD-1 monoclonal antibody for cancer therapy. Acta Pharmacol Sin. 2021 Jan;42(1):142-148.
[6] Zhang C, Lin R, Li Z, Yang S, Bi X, Wang H, Aini A, Zhang N, Abulizi A, Sun C, Li L, Zhao Z, Qin R, Li X, Li L, Aji T, Shao Y, Vuitton DA, Tian Z, Wen H. Immune Exhaustion of T Cells in Alveolar Echinococcosis Patients and Its Reversal by Blocking Checkpoint Receptor TIGIT in a Murine Model. Hepatology. 2020 Apr;71(4):1297-1315.
[7] Jebbawi F, Bellanger AP, Lunström-Stadelmann B, Rufener R, Dosch M, Goepfert C, Gottstein B, Millon L, Grandgirard D, Leib SL, Beldi G, Wang J. Innate and adaptive immune responses following PD-L1 blockade in treating chronic murine alveolar echinococcosis. Parasite Immunol. 2021 Aug;43(8):e12834.
[8] Moazeni M, Hosseini SV, Al-Qanbar MH, Alavi AM, Khazraei H. In vitro evaluation of the protoscolicidal effect of Eucalyptus globulus essential oil on protoscolices of hydatid cyst compared with hypertonic saline, povidone iodine and silver nitrate. Journal of Visceral Surgery. 2019;156(4):291-5.
